# Role of cervical vestibular evoked myogenic potentials (cVEMP) and auditory brainstem response (ABR) in the evaluation of vestibular schwannoma^☆^

**DOI:** 10.1016/j.bjorl.2016.04.003

**Published:** 2016-04-28

**Authors:** Deepa Aniket Valame, Geeta Bharat Gore

**Affiliations:** T.N. Medical College & BYL Nair Hospital, Department of Audiology and Speech Therapy, Mumbai, India

**Keywords:** Cervical vestibular evoked myogenic potentials, Auditory brainstem response, Contralateral effect, Neurofibromatosis, Potenciais evocados miogênicos vestibulares cervicais, Resposta auditiva evocada do tronco cerebral, Efeito contralateral, Neurofibromatose

## Abstract

**Introduction:**

Cervical vestibular evoked myogenic potentials (cVEMP) can assess the integrity of the inferior vestibular nerve thereby promising to be a useful tool in the audiological test battery to diagnose vestibular schwannoma.

**Objective:**

To ascertain the utility of cVEMP in diagnosis of vestibular schwannoma in conjunction with the ABR and to evaluate whether the size of lesion has any effect on the cVEMP measures.

**Methods:**

Case-files of 15 known cases of vestibular schwannoma whose pure tone audiometry, auditory brainstem response (ABR), cVEMP and radiological investigation findings were available, were included in the study. Patients were categorised as large or small tumours based on the size. The absolute and inter-peak latencies of ABR, amplitudes of waves V and I, and inter-aural latency difference of wave V of ABR; and latency of P1 and N1 of cVEMP and amplitude of P1–N1 complex were considered in the study.

**Results:**

There were eight large and nine small tumours. All the patients with large tumours showed significant severity of hearing loss whereas only three out of nine patients with small tumours showed severe to profound deafness in the affected ear. The rest showed hearing status ranging from normal hearing sensitivity to moderate hearing loss. Most of the patients with large tumours showed complete absence of ABR in the affected ears with no identifiable wave-peaks. ABR in small tumours exhibited delayed III–I and delayed V–I interpeak latency interval (IPL). Four out of five patients with large unilateral tumours revealed contralateral effects of reduced amplitude or absence of cVEMP. On the contrary, six out of eight unilateral small tumours showed a normal cVEMP response in the contralateral ear. Both the patients with NF2 in the present study demonstrated cVEMP abnormalities.

**Conclusion:**

ABR and cVEMP, when used in combination, can be of immense use in identification of neuro-otologic conditions such as vestibular schwannoma and bilateral tumours in NF2. In the evaluation of unilateral vestibular schwannoma, abnormal contralateral findings of cVEMP and ABR are strongly indicative of the tumour size >2.5 cm. In unilateral severe to profound loss wherein ABR in poorer ear cannot give information of site-of-lesion, cVEMP can help in the differentiation.

## Resumo

**Introdução:**

Os potenciais evocados miogênicos vestibulares cervicais (cVEMP) podem avaliar a integridade do nervo vestibular inferior, prometendo assim ser uma ferramenta útil na bateria de testes audiológicos para o diagnóstico de schwannoma vestibular.

**Objetivo:**

Determinar a utilidade de cVEMP no diagnóstico de schwannoma vestibular em conjunto com PEATE e avaliar se o tamanho da lesão tem qualquer efeito sobre as medidas do cVEMP.

**Método:**

Quinze casos conhecidos de schwannoma vestibular cujos achados de audiometria tonal pura, Potencial evocado auditivo de tronco encefálico (PEATE), cVEMP e investigação radiológica estavam disponíveis foram incluídos no estudo. Os pacientes foram classificados como portadores de tumores grandes ou pequenos. As latências absolutas e interpico de PEATE as amplitudes das ondas V e I e a diferença de latência interaural da onda V da PEATE e a latência de P1 e N1 de cVEMP e amplitude do complexo P1-N1 foram consideradas no estudo.

**Resultados:**

Havia oito tumores grandes e nove pequenos. Todos os pacientes com tumores grandes apresentavam perda auditiva grave enquanto apenas três dos nove pacientes com pequenos tumores apresentaram surdez grave a profunda na orelha acometida. O restante apresentou audição que variou de normal a perda auditiva moderada. A maioria dos pacientes com tumores grandes demonstrou ausência completa de PEATE nas orelhas acometidas sem picos de onda identificáveis. O PEATE em tumores pequenos apresentou Intervalo de Latência Interpico (ILI) tardia III-I e tardia V-I. Quatro em cada cinco pacientes com tumores grandes unilaterais revelaram efeitos contralaterais de amplitude reduzida ou ausência de cVEMP. Ao contrário, seis dos oito tumores pequenos unilaterais apresentaram resposta de cVEMP normal na orelha contralateral. Ambos os pacientes com NF2 no presente estudo demonstraram anormalidades na cVEMP.

**Conclusão:**

PEATE e cVEMP, quando utilizadas em combinação, podem ser úteis na identificação de condições neuro-otológicas como schwannoma vestibular e tumores bilaterais em NF2. Na avaliação de schwannoma vestibular unilateral, achados contralaterais anormais de cVEMP e PEATE são fortemente indicativos de tumor >2,5 cm. Na perda unilateral grave a profunda, na qual o PEATE na orelha mais prejudicada não fornece informações do local da lesão, cVEMP pode ajudar na diferenciação.

## Introduction

Vestibular schwannomas are benign intracranial tumours arising from the schwann cells of the vestibulocochlear nerve. Most of these tumours arise from the inferior vestibular branch and more than 90% are unilateral sporadic tumours while the rest are bilateral schwannomas due to Neurofibromatosis type II (NF2).^1^ The gold standard for the identification of these tumours is the gadolinium enhanced MRI which can identify even small tumours few millimetres in size. The audiological battery that was found most sensitive in the identification of these tumours using clinical decision analysis method includes the auditory Brainstem Response Audiometry (ABR) and Acoustic Reflex Combined (ARC), i.e. acoustic reflex threshold and reflex decay tests in combination.^2^ However, the audiological battery has its own limitations. The sensitivity of ABR decreases as the size of the tumour is less than one cm. Further click evoked ABR may miss out the tumours arising from the low frequency fibres of the vestibulocochlear nerve thereby decreasing its sensitivity.^3^ Also, the audiological tests are not useful if the affected ear has a hearing loss of 70 dB HL or greater. With the advent of MRI, thus the audiological test battery is not deemed very useful in the evaluation of vestibular schwannoma. One recent addition to the armamentarium of an audiologist in the last decade is the cervical Vestibular Evoked Myogenic Response (cVEMP).

cVEMP are short latency electromyogenic responses that are recorded from contracted sternocleidomastoid (SCM) muscle in response to very loud transient stimuli. These are thought to reflect momentary inhibition of the contraction of SCM due to loud sounds and are mediated by saccullocolic pathway.^4^ The outcome measures of cVEMP i.e. the latency of P1 and N1 waves, amplitude of P1–N1 complex and inter-aural amplitude ratio (IAR) are thought to reflect the functioning of saccule and/or inferior branch of vestibular nerve although presently the test cannot differentially diagnose between lesions of these two sites. As the inferior vestibular nerve is involved in neural lesions such as vestibular schwannoma, cVEMP can be a useful tool in the test battery. Further, the proposed pathway of cVEMP involves neural impulses from the inferior vestibular nerve reaching the inferior vestibular nucleus in the brainstem. The descending arc is supposedly via the medial vestibulospinal tracts (MVST) that supply the spinal accessory nerve to the effector muscle SCM.^5^ Thus lesions or tumours of the lower brainstem can affect the cVEMP pathway either in its ascending path (inferior vestibular nerve) or descending path (MVST). In conjunction with the ABR that reflects the synchronous discharge of onset-sensitive neurons from the cochlear nerve to the neurons in upper brainstem, cVEMP has a promising role in the evaluation for diagnosis of vestibular schwannoma.

Although the last decade has seen a surge of research papers in the clinical application of cVEMP in different clinical conditions, the role of this test in the test battery for identification of vestibular schwannoma is not as well-studied as the ABR or immittance testing. Further, if the size of the vestibular schwannoma has any effect on the cVEMP has not been explored. Hence clinical studies across various clinics involving measurement of cVEMP in known cases of vestibular schwannoma will help us to better understand what will be the role of this quick, non-invasive tool that can be performed on standard auditory evoked potential equipment, in the audio-vestibular diagnosis of these lesions.

To this end, this observational study was conducted to ascertain the utility of cVEMP in diagnosis of vestibular schwannoma in conjunction with the ABR. Further, descriptive statistics was used to evaluate whether the size of lesion has any effect on the cVEMP measures.

## Methods

The study was approved by the ethical committee set up by the university and methodology was in strict adherence to the approved protocol. Known cases of vestibular schwannoma that had reported for audiological evaluation were tested using the ABR and cVEMP. Fifteen diagnosed cases of vestibular schwannoma (17 ears) in the period from May 2012 to May 2014, whose pure tone audiometry, ABR, cVEMP and radiological investigation findings were available, were included in the study. Thirteen patients had unilateral sporadic schwannoma while two were Neurofibromatosis type II (NF2) patients with bilateral tumours. Patients with conductive pathology were excluded. Each patient's pure tone audiogram (PTA), click evoked ABR and cVEMP results were documented. PTA was carried out using Interacoustics AC 40 dual channel audiometer with TDH-39 earphones housed in MX 41-AR cushions. ABR and cVEMP were recorded using IHS Smart EP (Florida, USA). ABR was recorded using 100 μS clicks presented via inserts and responses were recorded from the non-inverting electrode on the forehead, inverting electrodes on the mastoids and the palm serving as the ground. cVEMP was monaurally recorded from electrode placed over contracted SCM using 500 Hz tone burst of rarefaction polarity presented at 80 dBnHL at a repetition rate of 5/s. The response was filtered from 10 to 1000 Hz and amplified 5000 times. Two runs of two hundred sweeps were recorded. cVEMP was carried out in sitting position. Unilateral SCM contraction was achieved by head rotation to the side contralateral to acoustic stimulation. Participants were instructed to bend down their heads by 30 degrees and then turn their head completely to one side thereby maintaining sustained contraction of SCM. Shoulder movements were discouraged. Patients were given a break after every run to avoid muscle fatigue. Two runs were recorded to ensure for intra-test reliability.

The absolute and inter-peak latencies of ABR, amplitudes of waves V and I and inter-aural latency difference of wave V of ABR; and latency of P1 and N1 of cVEMP and amplitude of P1–N1 complex were considered in the study.

## Results

The utility of cVEMP indices in conjunction with ABR indices in 17 ears with vestibular schwannoma is discussed to highlight the role of this test in the test battery. Of the fifteen cases reviewed, thirteen had unilateral sporadic vestibular schwannoma while two had Neurofibromatosis type 2 (NF2). The lesion was seen on the left ear in 6 cases and right ear in 7 cases while bilateral lesions were seen in the 2 cases with NF2. The age of the patients ranged from 19 years to 68 years with a mean age of 43.6 years.

Nine tumours were classified as small and eight as large based on their size as estimated from MRI scans. Tumours ≥2.5 cm were defined as large in this study while those <2.5 cm were defined as small. The tumour size varied from 5.4 mm to 5.0 cm.

Table 1 shows the severity of loss, presence/absence of ABR abnormality in ipsilateral and contralateral ear and presence/absence of cVEMP abnormality in ipsilateral and contralateral ear in these patients with unilateral vestibular schwannomas.

**Table 1:** Details of participants.

Identifier	Tumour side	Tumour size	PTA severity Affected side	ABR Ipsilateral ear	ABR Contralateral ear	VEMP Ipsilateral ear	VEMP Contralateral ear
1 VG	Left	Large	Profound	Absent	Mass effect	Absent	Absent
2 BJ	Left	Large	Severe	Absent	Mass effect	Reduced amplitude	Reduced amplitude
3 SG	Right	Large	Profound	Absent	Mass effect	Absent	Reduced amplitude
4 SD	Left	Large	Profound	Absent	Mass effect	Absent	Delayed latency, reduced amplitude
5 VV	Right	Large	Severe	Absent	NAD	Absent	NAD
6 RD	Right	Small	Severe	Absent	NAD	Poor morphology, delayed latencies and reduced amplitude	NAD
7 KK	Right	Small	Mild loss at low frequencies	Only wave I seen	NAD	Delayed latency, reduced amplitude	NAD
8 RV	Left	Small	Moderately severe	Delayed IPL. Absent high rate ABR	NAD	Absent	NAD
9 LK^*^	Left	Small	Normal hearing sensitivity	Delayed IPL	NAD	NAD	Reduced amplitude
10 KR	Right	Small	Normal hearing sensitivity	Delayed IPL	NAD	Delayed latency	NAD
11 PK	Left	Small	Moderate loss sloping to profound loss	Delayed IPL	NAD	Delayed latency, reduced amplitude	NAD
12 PG	Right	Small	Mild loss with 4 K dip	Delayed IPL	NAD	Delayed latency, reduced amplitude	Reduced amplitude
13 NM	Right	Small	Normal hearing sensitivity sloping to severe loss	Absent	Absent	Absent	NAD
14 DD	Right	Large	Profound	Absent	NA	Absent	NA
15 DD	Left	Large	Moderately severe rising to mild hearing loss	Amplitude of wave V < Wave I, Abnormal stacked ABR	NA	Absent	NA
16 LL	Right	Large	Severe hearingloss	Absent	NA	Absent	NA
17 LL	Left	Small	Normal hearing sensitivity sloping to moderate	Absent	NA	Delayed latency, reduced amplitude	NA

NA, Not applicable as the patients have bilateral tumours (NF2).

The above data reveals that all the patients with large tumours (100%, *n* = 8 ears) showed significant severity of hearing loss whereas only three out of 9 (33.33%) patients with small tumours showed severe to profound deafness in the affected ear. The rest showed hearing status ranging from normal hearing sensitivity to moderate hearing loss. Thus the size of the tumour does appear to affect the severity of hearing loss. This is consistent with the findings reported by Shih et al. who demonstrated a linear correlation between tumour size and deterioration of pure tone thresholds.^6^

Most of the patients with large tumours (87.5%, *n* = 7) showed complete absence of ABR in the affected ears with no identifiable wave-peaks. The only ear (DD, left ear) classified to have a large tumour but with ABR present was a tumour of size 2.8 × 2.3 cm, thus it was borderline large. On the contrary, absence of ABR was noted only in 33.33% (*n* = 3) patients with small tumours of which one showed wave I was spared. Eggermont, Don & Brackmann also report that only four of their forty three patients with small tumours showed absence of ABR.^7^ Thus absence of ABR is rare but possible even in few small tumours considering that factors like site of tumour, its consistency and vascularity may all affect results. The rest of ABRs in small tumours of the present study were characterised by delayed III–I and delayed V–I interpeak latency interval (IPL). One patient showed absence of peaks when high stimulus repetition rate was used. These indices have shown to be diagnostic in identifying tumours in the previous studies too.^6^, ^7^

All large tumours affected the ABR of contralateral ear in terms of reduced amplitude of latter waves, delayed latencies of latter waves and absence of wave V in one patient. Musiek and Kiebbe^8^ reported contralateral abnormalities of ABR in more than 70% of patients with tumours >3 cm and the most useful indicator was delayed V–III interpeak latency interval (IPL). Shih et al.^6^ studied thirty patients with vestibular schwannoma. They recommend that prolonged V–III IPL and wave V latency in contralateral ear, with prolonged III–I IPL in ipsilateral ear should be interpreted as a tumour >2 cm. They strongly advocated that when ipsilateral as well as contralateral abnormal parameters are considered for diagnosis, the predictive value of ABR in identification of tumour as well as its size increases. This is typically attributed to the mass effect of the lesion that pushes or rotates the brainstem towards the opposite side causing compression of the generators of the latter wave peaks of the ABR on the contralateral side. However other investigators refute this finding as large meningiomas with similar brainstem shift do not lead to equivalent abnormality on ABR. Musiek and Kiebbe^8^ say that compression of ipsilateral lateral lemniscus nuclei cause desynchronization of fibres responsible for wave V. The contralateral fibres also show desynchronization after their decussation leading to contralateral effect on ABR.

Except one patient (LM) with a small tumour, all patients (88.88%) in the present study revealed abnormality of cVEMP on the affected side irrespective of the size of tumour or severity of hearing loss. Seven out of eight ears with large tumours (87.5%) led to complete absence of cVEMP waveform whereas one showed severely diminished amplitude. The cVEMP was absent in two small tumours (22.2%), showed delayed latency in one patient (11.1%) and both delayed latency and reduced amplitude in five small tumours (55.5%). This is in accord with the findings of Murofuschi et al.^9^ who reviewed charts of 62 patients with acoustic neuromas and reported absence of cVEMP or decreased amplitude in 77% patients. Chen et al.^10^ also reported that eight of his nine patients with cerebellopontine angle (CPA) tumours showed affected cVEMP. They also stated that before surgery, cVEMP test can be used to predict the nerve of origin and to formulate the best surgical approach. After surgery, the test can be used to define the nature of the tumour (compressing or infiltrating the nerve) and disclose the residual function of the inferior vestibular nerve. One patient in the present study with a small tumour (LM) consistently showed a robust waveform on the affected ear and absent response in the opposite ear. This finding cannot be explained.

## Discussion

One of the aims of the present study was to determine if large vestibular schwannoma caused cVEMP to be abnormal when the opposite or unaffected ear was tested. As the descending pathway of cVEMP courses through the lower brainstem, it is possible that large tumours that have displaced/compressed the brainstem to the opposite side will show abnormalities in the response in contralateral ear, as evident on the ABR. It was observed that four out of 5 patients (80%) with large unilateral tumours revealed contralateral ear effects of reduced amplitude or absence of cVEMP. On the contrary six out of eight unilateral small tumours (75%) showed a normal cVEMP response when the contralateral ear was tested/stimulated. One patient (PG) showed a contralateral effect present whereas as mentioned previously LM showed paradoxical results of absent contralateral and normal ipsilateral cVEMP response (4 ears with of the two NF2 patients have not been considered here as contralateral effects cannot be estimated in bilateral tumours).

The high proportion of large tumours showing abnormal response when the opposite ear was tested could be due to the mass effect of the lesion on the contralateral inferior vestibular nuclei or the descending MVST in the brainstem. To the best of the researcher's knowledge there are no previous reports of this finding. Thus this study highlights an important use of the cVEMP in conjunction with ABR: not only detect acoustic tumours but also to estimate the approximate size based on the findings in the contralateral ear. Presence of bilateral abnormalities on the cVEMP (absence of response or reduced amplitude) and bilateral ABR abnormalities (especially prolonged V–III IPL or absent wave V) in case of unilateral hearing complaints is strongly suggestive of vestibular schwannoma >2.5 cm based on this study. This needs to be corroborated based on findings in larger number of cases.

Itoh et al. report of an interesting application of the combined use of ABR and cVEMP to differentiate between upper brainstem vs. lower brainstem lesions based on their study of thirteen patients.^11^ Patients with upper brainstem lesions showed cVEMP spared but ABR abnormal whereas those with lower brainstem lesions showed abnormalities on both the measures.

cVEMP is especially a useful tool in differential diagnosis of site of lesion when the affected ear has a severe to profound severity of hearing loss wherein the ABR and acoustic reflex testing are rendered to be of no use as they need residual hearing for their diagnostic utility. Absent ABR in a severe to profound loss leads to confounding results as the response could be absent either owing to the retrocochlear lesion or due to severity of cochlear loss. Since cVEMP does not need residual hearing as a pre-requisite for its elicitation, cVEMP findings in such cases can be crucial to identification of retrocochlear pathology. If a patient with unilateral severe or profound SNHL and absent ABR shows normal cVEMP response we can rule out the involvement of inferior vestibular nerve which is the site of vestibular schwannoma. However if cVEMP is absent too, radiological investigations and medical diagnoses are warranted.

### NF2

Both patients with NF2 (DD, LL) showed bilateral vestibular schwannoma with one ear showing larger tumour than the other. DD had a 4.8 × 2.8 cm tumour in the right ear and a 2.8 × 2.3 cm tumour in the left ear. Her ABR was grossly abnormal with no identifiable waveforms on the right ear and a near-normal ABR in the left ear with absolute and inter-peak latencies within normal limits but amplitude of wave V < Wave I. She was then taken up for tone-burst evoked stacked ABR in the left ear. In this procedure, a modified version of the derived-band procedure given by Don et al.; ABR was elicited in response to tone-burst stimuli of frequencies 500 Hz, 1000 Hz, 2000 Hz and 4000 Hz at 80 dBnHL. The waveforms were aligned for wave peak V and stacked to get a stacked ABR. The amplitude of the stacked ABR was 0.7 μV which was significantly reduced compared to the norms obtained on the same instrument. cVEMP was absent in the right ear and reduced in amplitude in the left ear with smaller tumour.

LL was referred from another centre for ABR and cVEMP. His MRI revealed a large lesion on the left side with a mass effect on the right side. Our testing, however, showed absence of cVEMP on the right side and delayed latency with reduced amplitude in the left ear. ABR was bilaterally absent in this patient. This immediately warned us about the probable right-left confusion on the MRI. On further evaluations it was confirmed that the patient was a case of NF2 with bilateral tumours, right being larger than the left.

Both the patients with NF2 in the present study demonstrated cVEMP abnormalities. Contrary to this, Wang et al.,^12^ reported that NF2 tumours more commonly originated from the superior vestibular nerve than the inferior vestibular nerve and infiltrated the cochlear nerve more than the inferior vestibular nerve. Only one of their seven patients demonstrated abnormal cVEMP as opposed to 77% of 14 ears that displayed abnormal caloric response mediated by the superior vestibular nerve.

## Conclusions

ABR and cVEMP, when used in combination, can be of immense use in identification of neuro-otologic conditions such as vestibular schwannoma and bilateral tumours in NF2. In the evaluation of unilateral vestibular schwannoma, abnormal contralateral findings of cVEMP and ABR are strongly indicative of the tumour size >2.5 cm. In unilateral severe to profound loss wherein ABR in poorer ear cannot give information of site-of-lesion, cVEMP can help in the differentiation.

## Conflicts of interest

The authors declare no conflicts of interest.
